# Vaginal microbiota: Potential targets for vulvovaginal candidiasis infection

**DOI:** 10.1016/j.heliyon.2024.e27239

**Published:** 2024-03-02

**Authors:** Yufei Wang, Zhaoxia Liu, Tingtao Chen

**Affiliations:** a Department of Obstetrics and Gynecology, The Second Affiliated Hospital of Nanchang University Jiangxi Medical College, No.1299, Xuefu Avenue, Honggutan District, Nanchang City, Jiangxi Province, China; b Queen Mary School, Jiangxi Medical College, Nanchang University, No.1299, Xuefu Avenue, Honggutan District, Nanchang City, Jiangxi Province, China; c School of Pharmacy, National Engineering Research Center for Bioengineering Drugs and the Technologies, Institute of Translational Medicine, Jiangxi Medical College, Nanchang University, No.1299, Xuefu Avenue, Honggutan District, Nanchang City, Jiangxi Province, China

**Keywords:** Vulvovaginal candidiasis, Probiotic, Postbiotic, Prebiotic, Synbiotic, Vaginal microbiota transplantation

## Abstract

Vulvovaginal candidiasis (VVC) is the second most common cause of vaginal infection globally after bacterial vaginosis (BV) and associated with adverse reproductive and obstetric outcomes, including preterm delivery, sexually transmitted infections and pelvic inflammatory disease. Although effective control of VVC is achievable with the use of traditional treatment strategies (i.e., antifungals), the possibility of drug intolerance, treatment failure and recurrence, as well as the appearance of antifungal-resistant *Candida* species remain critical challenges. Therefore, alternative therapeutic strategies against VVC are urgently required. In recent years, an improved understanding of the dysbiotic vaginal microbiota (VMB) during VVC has prompted the consideration of administering -biotics to restore the balance of the VMB within the context of VVC prevention and treatment. Here, we aim to summarize the current evidence of the anti-*Candida* effects of probiotics, postbiotics and synbiotics and their potential use as an alternative/complementary therapy against VVC. Additionally, this review discusses advantages and challenges associated with the application of -biotics in VVC to provide guidance for their later use. We also review new developments in VVC therapy, i.e., vaginal microbiota transplantation (VMT) as an emerging live biotherapeutic therapy against VVC and discuss existing shortcomings associated with this nascent field, expecting to stimulate further investigations for introduction of new therapies against VVC.

## Graphical abstract

**Figure:**
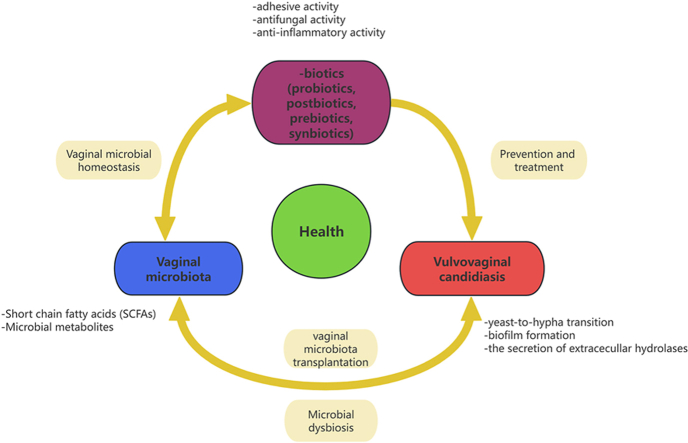

## Glossary

**Probiotics**: Live microorganisms which when administered in adequate amounts confer a health benefit on the host
**Prebiotics**: a substrate that is selectively utilized by host microorganisms conferring a health benefit
**Synbiotics**: the mixture of prebiotics and probiotics which can show a synergistic beneficial effect on host health
**Postbiotics**: bioactive soluble factors (products or metabolic byproducts) produced by live probiotic microorganisms or released after the cell rupture that confer any physiological benefit to the host
**Vulvovaginal candidiasis**: a symptomatic vaginitis (inflammation of the vagina and/or vulva) caused by infection with a *Candida* yeast

## 1 Introduction

Vulvovaginal candidiasis (VVC) is characterised by vaginal and vulval infection in the lower female genitalia and initiated by *Candida* species [1]. As the second leading cause of vaginitis after bacterial vaginosis (BV) worldwide, 70%–75% of all women in their reproductive years will suffer at least one episode of VVC [2]. Reduced quality of life and social relations including sexual activity were reported by infected women due to vulvar pruritus, burning and cottage‐cheese‐like vaginal discharge [3]. In addition, VVC is related to an increased risk of reproductive and obstetric complications such as intra-amniotic infection, preterm delivery and low-birth-weight infants [4]. Although antifungal drugs (e.g., azoles) are still the mainstream therapy for VVC, their therapeutic value is limited due to potential toxicity, the appearance of drug-resistant strains and high recurrence rates [5]. Therefore, a complementary or alternative therapy to overcome shortages of antifungals and effectively treat VVC is urgently required in clinical work.

The vaginal microbiota (VMB) constitutes about 9% of the total human microbiota and maintains a mutualistic relationship with the host [6]. Abundant evidence suggest that healthy reproductive women are characterized by *Lactobacillus*‐dominated vaginal microbial communities, which defend the vaginal milieu against infections by pathogens through the competitive inhibition, the secretion of antimicrobial compounds and the regulation of host immune responses [7]. Nevertheless, the disturbance of the vaginal ecosystem may lead to a dysbiotic VMB with lactobacilli depletion and the overgrowth of pathogens, causing vaginal infections such as BV, trichomoniasis (TV), aerobic vaginitis (AV) and VVC, and increasing the risk of obstetric complications and sexually transmitted infections (e.g., HIV) [7,8]. Therefore, a growing body of research has proposed that interventions designed to restore the vaginal microbial balance may be therapeutically feasible in the treatment of VVC.

Probiotics are live microorganisms that provide a health benefit for the host when given in adequate amounts [9]. Over the recent years, probiotics (e.g., *Lactobacillus, Bifidobacterium* and *Streptococcus* preparations) have been applied in the prophylaxis and management of several disorders such as irritable bowel syndrome (IBS), hypertension, vaginal infections and cancers [10,11]. For example, the supplementation with live *Lactobacillus* has been widely reported to improve the vaginal health of BV patients through restoring the normal vaginal microbiotic environment [12]. Since mounting evidence showed the ability of probiotics to suppress virulent traits of *Candida* spp., produce antifungal peptides, and modulate local cervicovaginal mucosal immune responses, probiotics have also been proposed to improve the therapeutic outcome of VVC [13]. In addition, the effectiveness of probiotics is enhanced by postbiotics (substances released via the metabolic activity of microorganisms) and prebiotics (products that improve the growth and/or activity of helpful microbes), which have also been applied in the management of VVC [14]. In this context, this review focuses on limitations of traditional methods in VVC therapy, evaluates the clinical efficacy of the use of -biotics for VVC and illustrates their potential risks. In addition, we also present emerging therapeutic strategies such as VMT and discuss its prospects for VVC treatment [15].

## 2 VMB plays an important role in maintaining vaginal health

According to high‐throughput 16S rRNA gene sequencing studies, the VMB of asymptomatic women can be classified into five microbial community state types (CST), of which four are dominated by *Lactobacillus* species (CST-I; *L. crispatus*, CST-II; *L. gasseri*, CST-III; *L. iners*, CST-V; *L. jensenii*); CST-IV, on the other hand, is characterised by a polymicrobial mixture of facultative and obligate anaerobes including *Prevotella, Dialister*, *Fannyhessea*, *Gardnerella* and *Mobiluncus* [6]. Although the community of the VMB varies among women in different regions, a *Lactobacillus*-dominant configuration tends to be associated with healthy vaginal states. A key protective mechanism is the production of lactic acid via the fermentation of glycogen by-products, which contributes to acidifying the vagina to a pH 3.5–4.5 [16]. Lactic acid has been proved to suppress numerous reproductive tract pathogens, such as *Neisseria gonorrhoeae*, *Candida* spp. and BV-associated bacteria [17]. Additionally, lactic acid also exerts immunomodulating effects through directly inhibiting pro‐inflammatory mediators IL‐6, IL‐8 and TNFα, and activating the interleukin 23 (IL-23)/IL-17 T lymphocyte pathway [6,18]. Other postulated mechanisms by which *Lactobacillus* strains impose a positive impact on vaginal health include the secretion bacteriocin, which kills a wide range of potentially hazardous microorganisms based on the permeabilisation of the target cell membrane [19]. Furthermore, although different *Lactobacillus* species vary greatly in thier capacity to adhere to host cells, many *Lactobacillus* species exhibit a strong effect of competitive exclusion by competing with pathogens for receptor-binding sites as well as available nutrients, resulting in the exclusion and rejection of pathogens [20]. For example, *L*. *brevis*, *L. salivarius* and *L. gasseri* have showed their ability to reduce more than 50% in the adherence of *Gardnerella vaginalis* and *Candida albicans* to epithelial cells [21]. Studies also showed that *L. crispatus*‐dominated VMB are able to enhance the integrity of the vaginal mucosa, which decrease susceptibility to infectious agents such as HIV [22].

The vaginal microbial niche is a dynamic micro-environment subjected to constant fluctuations throughout a woman's lifetime [6]. Several predisposing factors, such as hormonal alterations, diabetes, uncontrolled administration of antibiotics, menstrual hygiene behaviours, sexual activity and vaginal douching, can significantly disrupt the VMB composition, contributing to the reduction of *Lactobacillus* spp. and, thus, vaginal dysbiosis, which leads to complicated vaginal infections including BV, TV and VVC [8]. For example, BV is featured by a shift in VMB with the protective lactobacilli depletion and a concomitant overgrowth of facultative and anaerobic organisms [7]. In addition, there are various non-infectious gynecological conditions associated with vaginal dysbiosis [6]. Studies have found that BV-associated VMB are more common in patients with tubal infertility than other causes of infertility [23].^23^Al-Memar M et al. reported that vaginal *Lactobacillus* spp. consumption and a higher diversity of the microbial community are related to an elevated risk of first-trimester miscarriage [24]. Miscarriage and preterm labour are considered to be associated with intrauterine infections caused by potential pathogens ascending from the vagina, or with immune responses to pathogens triggering pro-inflammatory cascades, which ultimately affect endometrial receptivity and implantation [25,26]. The overgrowth of *Candida* species may activate IL-6 receptors and TLR-2 in the placenta thus increase NF-κB level, leading to the intrauterine infection in the newborn [27]. Therefore, as a balanced VMB plays a major role in improving vaginal wellness, women with gynecological conditions may benefit from interventions that restore normal microbial composition and structure.

## 3 VVC: the pathogenesis and currently available treatment regimens

VVC is a fungal infection of the mucosa lining the female genital tract as a result of the invasion of *Candida* species. About 75% of all women of reproductive age will suffer at least one episode of VVC with predisposing factors such as the use of antibiotics, sexual activities and the ingestion of oral contraceptives. And 5%–8% develop recurrence vulvovaginal candidiasis (RVVC) characterised by four or more episodes over a period of 12 months, which decreases patients’ quality of life, self-esteem and confidence, coupled with unbearable symptoms, and contribute to a high medical burden [2,28]. Specifically, *C. albicans* is responsible for more than 90% of VVC conditions, whereas the non-*albicans Candida* (NAC) species, including *C. glabrata*, *C. krusei*, *C. tropicalis* and *C. parapsilosis*, are responsible for the remaining 10% [29]. Under normal circumstances, yeast forms of *Candida* spp. are found as a portion of the normal VMB and colonise the vaginal lumen asymptomatically, which may attribute to the balance between *Candida* growth and immune responses [30]. However, morphogenesis-stimulating environments (e.g., absence of nutrients, enhanced vaginal pH and microbial destruction) trigger yeast-to-hypha transition by activating the cAMP/PKA pathway, thus up-regulating the transcriptional level of hypha-associated genes (*HWP1 and HYR1*) [31]. The hyphal form has been considered to be more invasive than the yeast form, allowing *Candida* to penetrate mucosal barrier and cause tissue damage [32]. Enhanced fungal recognition mediated by pattern recognition receptors (PRRs), epithelium invasion and the secretion of extracellular hydrolases (candidalysin, lipase and secreted aspartyl proteinases) elicit the production of pro-inflammatory mediators such as IL-1β and S100A8/9 alarmins in the vaginal epithelial cells via NLRP3 inflammasome activation [29]. These cytokines and chemokines result in polymorphonuclear leukocyte (PMNs) chemotaxis to the vaginal lumen, which attempt to eliminate *Candida* species. The activation of complement system can facilitate the phagocytosis through the surface opsonization [33]. However, *Candida*. may employ many strategies to evade host defence such as inhibiting complement activation and phagolysosome formation, and modulating T cell function [33]. Moreover, the ability of *Candida* spp. to form biofilms reduces the penetration of host immune cells through the matrix, making them intrinsically resistant to host immune responses, such as phagocytosis by macrophages and PMNs. Therefore, insufficient inflammatory responses and the tissue damage caused by *Candida* contribute to the immunopathogenic process and symptoms of VVC, including itching, soreness, dysuria, dyspareunia and cottage cheese-like vaginal discharge [34].

Currently, there are various treatment approaches for VVC, based on uncomplicated (*C*. *albicans-*induced VVC with sporadic attacks and slight symptoms) or complicated (NAC-initiated RVVC with severe symptoms) cases. Classic medications principally involve antifungal agents in the form of prescribed oral agents, over-the-counter topical cream and intravaginal suppositories [35]. Azoles (e.g., ketoconazole, itraconazole and fluconazole) are suitable for the treatment of almost all cases of uncomplicated VVC [35]. Basically, azoles inhibit fungal activities by suppressing the synthesis of ergosterol, the major component of fungal cell walls, via the suppression of cytochrome P450 (encoded by *ERG11*)-catalysed 14α-demethylation of ergosterol precursors [36]. Other antifungals, such as polyenes (e.g., nystatin and amphotericin B) and echinocandins (e.g., caspofungin and micafungin), function by binding to ergosterol within the fungal cell walls, which causes the leakage of intracellular components including potassium, magnesium and sugars through pore formation thus results in fungal death [37]. It is not determined if oral or topical formulations are better for treating VVC, and no single agent seems to be obviously better than others [38]. The present therapeutic suggestions against VVC by the American Centers for Disease Control and Prevention (CDC) consist of a 3–7 day topical azole therapy or a single dose of 150 mg of fluconazole administered orally [39]. Pregnant patients should only be given topical azoles for a maximum of 7 days in symptomatic hosts due to the potential risk of abortion or foetal abnormality associated with oral fluconazole [40]. Compared to most episodic or sporadic VVC, the treatment of women with complicated VVC needs a long-term-maintenance antifungal regimen (6 months) to ensure symptom remission [41].

Although antifungal agents have successfully been used for decades in the treatment of VVC, none has all the properties of an ideal drug [38]. And their efficacy is compromised due to low bioavailability, potential side effects and the appearance of drug-resistant strains [42]. The side effects of interventions with antifungals involve idiosyncratic hepatotoxicity, abdominal discomfort and nausea with the oral administration as well as vaginal burning or soreness with topical azoles [43]. In addition, the increasing availability and accessibility of over-the-counter antifungal medications as well as prolonged therapy may contribute to the appearance of drug-resistant strains. The main azole resistance mechanisms include the up-regulation of multi-drug transporter gene expression (*CDR1*, *CDR2 and MDR1)* and the alteration of the target enzyme via non-synonymous *ERG11* mutations [44,45]. Another mechanism of azole resistance involves the replacement of ergosterol with 14-α-methylfecosterol via mutations in the *ERG3* gene, which counteracts the destructive effects of azoles on the *Candida* cell membrane [46]. In recent years, VVC caused by NAC species has become more common, which tend to show intrinsic resistance to azoles and cause ineffective treatment [47]. Moreover, the quorum sensing between biofilm microorganisms, the expression of efflux pumps and the presence of persister cells in *Candida* biofilms are associated with low susceptibility to antifungals, which leads to a significant clinical challenge [48]. Sobel et al. (2004) found that there are still 9.2%, 26.8% and 57.1% of women who experience relapse at 6, 9 and 12 months, respectively, after therapy cessation since failure to thoroughly eradicate biofilms results in the continued presence of persistent cells [49]. Moreover, the current antifungal therapeutic strategies are not able to restore a balanced VMB, which is also associated with a great risk of recurrent infections. Therefore, an investigation of novel and alternative therapeutic strategies against VVC and RVVC is urgently needed to overcome the shortages of current antifungal therapies.

## 4 Administration of -biotics as a complementary/alternative treatment strategy for VVC

### 4.1 The potential of *Lactobacillus*-based probiotics in the treatment of VVC

*Lactobacillus* species are Gram-positive rods dominating the resident microbiota colonising the vagina and gastrointestinal tract, which have been widely used as probiotics to restore the state of vaginal eubiosis [50]. In recent years, *Lactobacillus* species have demonstrated a short-term and long-term beneficial effect against BV characterized by vaginal *Lactobacillus* depletion [51]. Since the decrease in *Lactobacillus* colonisation rates (<67.5%) and the alteration of dominant species (*L. crispatus* gradually replaced by *L. iners*) were also observed during VVC states, which lead to a higher vaginal pH thus increase the risk of *Candida* proliferation, *Lactobacillus*-based probiotics have been proposed as an alternative strategy to restore the VMB balance against VVC [52,53]. Kumherová et al. (2021) isolated 41 *Lactobacillus* strains from vaginal secretes of healthy women and evaluated their *in vitro* activity against *Candida* spp. Most *Lactobacillus* strains (particularly *L. crispatus*, *L. fermentum*, and *L. rhamnosus*) exhibited great auto-aggregation and anti-*Candida* activity, suggesting their role in the treatment of VVC [54,55]. Multiple animal model experiments and clinical studies have also assessed the *Candida* colonisation rate and VVC occurrence following oral or intravaginal *Lactobacillus* administration either alone or in various combinations. Different inhibitory effects have been observed for these *Lactobacillus* strains, demonstrating that treatment effects rely on the specific strain used, the timing of probiotic treatment and the dose administered [56].

The specific mechanism underlying the antifungal effect of *Lactobacillus* strains in the vagina has been extensively evaluated. (1) They reduce the initial adhesion and colonisation of *Candida* species through competing for adhesion sites and nutrients on mucosal surfaces, (2) they exhibit fungicidal or fungistatic effects via the production of secondary metabolites with antimicrobial activity, (3) they modulate the immune response[Fig. 1] [13,20]. Besides, the administration of probiotics in combination with antifungal drugs has a synergistic effect, which improves the susceptibility of *Candida* spp. to conventional therapy by reducing the expression levels of drug-resistant genes in *Candida* [57].

(a) The role of single strains in the prevention and treatment of VVC

*L. acidophilus* is one of the most common vaginal lactobacilli in health women, which has revealed the required probiotic properties and successfully applied in the treatment of urogenital infections [58]. The anti-*Candida* effects of *L. acidophilus* have been explored in *in vitro* and *in vivo* models. *Candida* cultivated together with *L. acidophilus* showed a lower metabolic activity compared with that of the control group due to inhibitory molecules secreted by lactobacilli, such as sodium butyrate [56,59]. *L*. *acidophilus* interferes with the ability of *Candida* in morphogenesis and biofilm formation by suppressing adhesion-related genes (*ALS3*) and transcriptional regulatory genes (*BCR1* and *CPH1*) expression, suggesting a role in reducing cellular damage and inflammation during VVC [60]. Rossoni et al. (2015) also observed the therapeutic role of *L. acidophilus* in the *Galeria mellonella* invertebrate host model. The authors discovered that the prophylactic administration of probiotics prevented *G. mellonella* from suffering from experimental candidiasis by stimulating host immune responses, such as an increase in haemocyte quantity and the production of antifungal peptides (e.g., galiomicin and gallerymicin) [61]. Similarly, the probiotic *L. acidophilus* supplement can enhance the clearance of *C. albicans* from mice with candidiasis and reduce tissue damage via modulating the cytokine profile, i.e., the down-regulating pro-inflammatory cytokines TNF-α, IL-12, and IL-6 [62].

Several clinical trials have reported the protective or therapeutic potential of *L. acidophilus* in the context of VVC. For example, Hilton et al. (1992) found that candidal colonisation and infection of women (aged 24–50 years) with rVVC were obviously decreased after 6 months of *L. acidophilus* administration [63]. Similarly, Williams et al. (2001) assessed the efficacy of the intravaginal application of *L. acidophilus* against VVC among HIV-positive women (aged 25–59 years) in a randomised controlled study. The probiotic group showed a lower risk of VVC and a prolonged median time to first attack of VVC compared with the control group [64]. These results suggested that *L. acidophilus* may be used to restore a normal VMB and become a potential complementary therapy for VVC. In another study, the drug resistance of *C. albicans* strains was reversed by *L. acidophilus* supernatant due to the inhibition of drug efflux proteins, which increased the sensitivity to antifungal drugs [65].

Other probiotics (e.g., *L. rhamnosus*, *L. delbrueckii* and *L. plantarum*) can also suppress the growth of major VVC-inducing strains, including *C. albicans*, *C. tropicalis*, *C. norvegensis*, *C. parapsilosis* and an emerging multi-resistant strain, *C. auris*, both *in vitro* and *in vivo* [[65], [66], [67]]. Moreover, *L. delbrueckii* is currently the only commercially available vaginal product of *Lactobacillus* (Inner Mongolia Shuang Qi Pharmaceutical Limited by Share Ltd.) which has been approved to treat vaginal dysbiosis for many years by interfering with the cytotoxic effects and colonisation of pathogens [68]. Therefore, single strains of probiotics have the potential to be used as adjunct bio-therapeutic agents to treat women with VVC.

(b) The role of probiotic combinations in the prevention and treatment of VVC

Numerous studies indicate that both oral and local combinations of probiotics can be efficient in the treatment of VVC. To date, the co-administration of two probiotic strains, *L. rhamnosus* GR-1 and *L. reuteri* RC-14, produces the most promising results at reconstituting and maintaining a normal VMB and has been used in the management of vaginal infections such as BV [69]. In 2003, a clinical study performed by Reid et al. indicated that the oral intake of the probiotics *L. rhamnosus* GR-1 and *L. reuteri* RC-14 is not only safe in healthy women (aged 19–46 years) but inhibits vaginal invasion by *Candida* yeasts [70]. In 2009, Martinez et al. performed a clinical trial with 55 women (aged 16–46 years) to evaluate the efficacy of the oral administration of *L. rhamnosus* GR-1 and *L. reuteri* RC-14 against VVC. Based on their results, probiotics remarkably reduced the amount of thick cheese-like vaginal discharge and the population of culturable yeasts compared to the placebo group [71]. To improve the understanding of underlying mechanisms of anti-*Candida* activity of probiotics, Martinez, R.C. et al. (2009) demonstrated that *L. rhamnosus* GR-1 and *L. reuteri* RC-14 inhibited the growth of co-incubated yeasts using a human vaginal epithelial cell line (VK2/E6E7) simulating VVC, and their cell-free supernatant (CFS) may up-regulate IL-8 and IP-10 secretion by VK2/E6E7 cells [72]. Wagner et al. (2012) showed that *L. rhamnosus* GR-1 and *L. reuteri* RC-14 suppressed the expression of NF-κB-related pro-inflammatory cytokines after incubation with *C. albicans* onVK2/E6E7 cells, suggesting their role in restricting the tissue damage mediated by excessive inflammation *in vivo* [73]. In 2015, Chew et al. were the first to explore if probiotic *Lactobacillus* strains have an antagonistic impact on the NAC species *C. glabrata*. Co-administration of *L. rhamnosus* GR-1 and *L. reuteri* RC-14 strains impeded the growth and biofilm formation of *C. glabrata in vitro* by forming co-aggregation and decreasing the expression of the biofilm-associated genes *EPA6* and *YAK1* [74]. Moreover, Kohler et al. (2012) demonstrated that after co-culturing with *C. albicans*, the probiotic combination can repress the target enzyme Erg11p and the ABC transporter Cdr1p, resulting in greater susceptibility of *Candida* cells to antifungal drugs [75]. Therefore, the use of *L. rhamnosus* GR-1 and *L. reuteri* RC-14 may become a promising alternative/complementary treatment option against VVC. On the other hand, the combined application of *L*. *crispatus* and *L*. *fermentum* significantly prevented the proliferation of *C*. *albicans in vitro* and in a murine VVC model [76]. The probiotic combination of *L. fermentum* and *L. acidophilus* also showed powerful *in vitro* inhibiting effects toward four *Candida* species (*C. albicans*, *C. glabrata*, *C. parapsilosis* and *C. krusei*). The effectiveness of probiotic supplements was subsequently confirmed in a clinical trial including 30 patients with VVC, with the result of relief from symptoms by 86.6% of patients and a long-term physiological defense [77].

Several clinical trials have demonstrated the beneficial effect of probiotic combinations co-administrated with antifungal drugs during VVC treatment. For instance, Ehrstrom, S. et al. (2010) suggested that conventional VVC therapy plus intravaginal administration of probiotics (*L gasseri* LN40, *L. fermentum* LN99, *L. casei* subsp. *rhamnosus* LN113 and *P. acidilactici* LN23) resulted in fewer recurrences (93% versus 83%) and a decrease in malodorous discharge compared with the placebo group [78]. Kovachev et al. (2015) also stated that a short course of antifungal therapy (fluconazole and fenticonazole) followed by a 10-day lactagyn treatment (*L. acidophilus, L. rhamnosus, S. thermophilus, L. delbrueckii* subsp. *bulgaricus*) contributed to a lower VVC recurrence rate 7 weeks after the cessation of therapy compared with using antifungals alone [79]. In a randomised, double-blind, clinical trial performed by Davar et al. (2016), 59 VVC women (the average age was 32.25) were orally treated with probiotic tablets (*L. acidophilus* and *B. longum*) or placebo for half a year after the initial treatment with oral fluconazole. The 6-month recurrence rate observed in the probiotic group was significantly decreased by 28.3% [80].

Vahedpoor Z et al. (2021) also investigated the action of vaginal probiotics (*L. acidophilus, L. plantarum, L. rhamnosus, L. gasseri*) and oral probiotics (*L. acidophilus, L. plantarum, L. fermentum, L. gasseri*) on 76 participants with VVC (aged 18–48 years) in a random, placebo-controlled trial. These patients were administered fluconazole supplemented with either probiotic capsules or a placebo. At 35 days after the intervention, the probiotics supplementation group reported an obvious reduction in VVC symptoms, such as less vaginal discharge, compared with the fluconazole plus placebo group [81]. In conclusion, compared with conventional antifungal drugs used alone, probiotics-supplemented treatments are more effective in increasing the short-term clinical remission rate and fungal clearance, and may result in a lower recurrence of VVC infections.

### 4.2 Yeast-based probiotics exert beneficial therapeutic effects against VVC

The past decades have seen a growing interest in the application of yeast-based probiotics. Since they have low sensitivity to most antibiotics, there is no need to describe their antibiotic resistance characteristics, and thus, they can be directly prescribed to patients treated with antibiotics compared to probiotics originated from bacteria [82]. Although numerous yeast species have probiotic properties, *Saccharomyces boulardii* and *S. cerevisiae* are the only probiotic yeasts commercially available. And *S. boulardii* is the first yeast to be used as a probiotic in human medicine, which has been recommended to prevent and treat intestinal disorders, such as diarrhoea related to antibiotics [83]. In 2010, *in vitro* studies performed by Murzyn et al. showed that *S. boulardii* cells and their extracts (e.g., capric acid) have inhibitory effects on the morphological transformation, colonisation and biofilm formation of *C. albicans.* This result was supported by the formation of co-aggregates and the down-regulation of *C. albicans virulence gene expression (HWP1*, *INO1* and *CSH1)* [84]. In other studies, *S. boulardii* suppressed the colonisation of *C. albicans* to epithelial cell lines and reduced elicited inflammation through inhibiting the pro-inflammatory cytokines IL-8 [85]. A prospective, randomised, comparative study carried out by Demirel et al. (2013) described the treatment of *S. boulardii* for VVC, which was supported by the reduction in *Candida* adhesion and invasive candidal infections in newborns [86]. Similarly, another trial conducted in preteen children used a probiotic mix containing *S. boulardii*, which impeded colonisation by *C. albicans.* [87] Moreover*,* the capric acid secreted by *S. boulardii* may enhance the activity of fluconazole and amphotericin B against *C. albicans* by inhibiting the function of the multi-drug resistance (MDR) transporter Cdr1p in excluding drugs, suggesting the synergistic potential of *S. boulardii* in combination with antifungal drugs against VVC [88].

Numerous investigations have emphasised beneficial effects of *S*. *cerevisiae*-based probiotics on BV, inflammatory bowel disease (IBD) and systemic candidiasis (*C. albicans* and NAC species) No severe adverse effects were detected, verifying the security and well-tolerability of this type of probiotics [[89], [90], [91]]. Pericolini et al. (2017) proved that the local administration of both living and inanimate *S. cerevisiae* shortened the course of VVC through speeding up the removal of *C. albicans* from the infected vagina of mice. This beneficial effect attributed to the obstruction of *Candida* adhesion to the mucosal surface via forming co-aggregates, which prevented tissue damage induced by *Candida* [91]. However, only the live yeast interferes with *Candida* virulence factors, such as yeast-to-hyphae transition via the inhibition of the transcription of hyphae-related genes (*HWP1* and *ECE1*), and the production of secretory aspartyl proteases (*SAP2* and *SAP6*), which are of great importance in the inflammasome-dependent inflammatory process of VVC [91,92]. In addition, *S. cerevisiae-*based probiotics play a role in preventing VVC through modulating the host immune response, such as reducing *C. albicans*-induced PMN influx and increasing their antimicrobial properties, i.e., the production of reactive oxygen species (ROS) and killing activity [93]. The role of *S. cerevisiae* in inhibiting virulence traits of several NAC species, including adhesion, morphological transition and biofilm formation, has been reported. It is also proposed that the advanced injection of probiotic yeasts *in vivo* prevents *Caenorhabditis elegans* models from gut colonisation by NAC strains and related inflammation [94]. In 2020, Stabile et al. initiated an experimental study to assess if the use of the Unilen Microbio+ (*S. cerevisiae*, melatonin and GLA-14) supplement therapy achieved better results than using clotrimazole alone among 40 women (aged 18–55 years) with uncomplicated VVC. Although both groups presented satisfactory clinical responses to the therapy, the recurrence rate in the clotrimazole-only group (40%) was twice as high as that in the group undergoing Unilen Microbio + therapy (20%) [95]. Additionally, increased *Lactobacillus* colonisation and a reduction in the number of PMN were observed in the VMB isolated from patients treated with Unilen Microbio+ [95]. Another study reported a synergistic effect between live *S. cerevisiae* and other probiotic species in the management of VVC by enhancing the viability of *L. rhamnosus* in an acidic environment [96]. Overall, these results indicate that probiotic yeasts are a promising alternative to antifungal therapy for VVC.

### 4.3 Potential challenges related the use of probiotics

Although numerous *in vitro* and clinical studies have been carried out to prove the physiological functions of probiotics in the clearance of *Candida* species, to date, no probiotics are commercially available to treat VVC/RVVC in the clinical practice. Because major medical regulatory authorities have regulated probiotics as dietary supplements rather than strictly controlled medications, there is a lack of standardised management strategies and quality monitoring for live microorganisms [97]. As a result, consumers are likely to obtain a useless product or one with different numbers of bacterial or yeast cells. In addition, many studies on probiotics neither used large sample sizes nor randomised, double-blinded and/or control groups. Therefore, the conclusion of these studies are questionable because of reduced statistical power and methodological limitations. Also, heterogeneity among studies cannot be ignored due to strain-specific benefits, patient variables (hormone states, age, genetic predisposition, sexual practice, vulva cleaning), the course of therapy and the type of probiotic tested (optimal dosage, formulation, mode of delivery) [98]. Again, the precise mechanism of probiotics is still unknown and needs further exploration. Therefore, it is of great importance to conduct more investigations with high-quality randomised controlled trials and to determine future research directions. Moreover, although no severe undesirable effects related to probiotic supplementation have been observed, several trials have reported mild or moderate gastrointestinal symptoms such as nausea and abdominal distention [99,100]. There is also a potential risk of probiotic-related bacteraemia and fungaemia in neonates, pregnant women, the elderly and immunocompromised patients [100]. Consequently, the safety of probiotic products should be guaranteed in the above at-risk populations through determining factors such as infectivity, pathogenicity, detrimental metabolic activity and potential gene transfer [9].

### 4.4 Opportunities and prospect of VMT as a novel live biotherapeutic approach against VVC

Currently, microbiome transplantation represents a promising therapy for various pathological conditions associated with dysbiosis, and most clinical evidence has been gained from successful faecal microbiota transplantation (FMT) [101]. FMT was firstly described in traditional Chinese medicine in the fourth century and referred to the introduction of gut microbiota isolated from faecal material of healthy donors into recipients’ GI tract to restore intestinal microbial diversity and normal bowel function [102]. Several studies have reported FMT as a mature therapy for various gastrointestinal conditions, such as recurrent *Clostridioides difficile* infections, pseudomembranous colitis, IBD and IBS [102,103]. Since using single probiotics or probiotic combinations orally or intravaginally to treat VVC patients produces mixed results, it is suggested that transplanting a microbial community may achieve a more effective clinical outcome [104]. Given the similarity between the gut and the vagina regarding physiological conditions and the pathogenesis of infections caused by the overgrowth of pathogens, researchers recently extended the probiotic therapy and put forward VMT as one of the promising treatments for VVC [105].

In brief, VMT involves the translocation of the total VMB community or probiotic strains with beneficial characteristics from the vaginal secretion of healthy women to recipients, aiming to construct a new vaginal micro-ecological balance [Fig. 2] [15]. In 1955, Gardner and Dukes first reported successful VMT procedures in humans by inducing BV in 11 out of 15 volunteers (73%) via direct transfer of vaginal material from infected women [106]. Based on these promising findings, a bio-molecular engineer at Johns Hopkins University, Laura Ensign, considered that introducing the entire VMB from a healthy vagina to patients with BV has therapeutic potential [107]. In 2019, Lev-Sagie et al. performed a clinical study to assess the clinical outcome of VMT in five patients (aged 27–47 years) with recurrent and antibiotic-non-responsive BV after 7 days of intravaginal antibiotic treatment. Four out of five patients reported an improved vaginal health in the long term (symptoms improvement, negative Amsel criteria and restoration of a normal *Lactobacillus*-dominated microbiota under the microscope) after 1–3 VMT sessions, and none of the five women experienced any adverse effects [108]. The results indicated that VMT intervention may achieve a better therapeutic outcome in BV as compared to using antibiotics alone. To exploring underlying mechanisms, Chen et al. (2021) reported that VMT protected rat models from recurrent vaginal dysbiosis via reducing pro-inflammatory cytokines such as IL-1β and TNFα in vaginal tissue and successfully restored the normal VMB [109]. Moreover, VMT has been reported to be safe and effective for normalizing neurodevelopment and the fecal microbiome in infants [110]. Therefore, it is suggested that VMT may act as a potential adjuvant therapy to other conventional treatments in alleviating vaginal disorders, such as the recurrent form of VVC.

Despite the promising therapeutic potential of VMT, this approach also has some limitations, such as ethical issues, inter-individual variability of the VMB, and the potential risk of infections or immunological rejection [15]. Although adverse effects associated with VMT have not been observed yet, risks of gynaecologic and obstetric complications induced by sexually transmitted pathogens, particularly antimicrobial-resistant microorganisms, cannot be excluded. Moreover, long-term effects of VMT remain unknown [111]. Therefore, it is necessary to improve safety precautions by establishing stringent criteria for VMT donor screening and selection [112]. For example, the frequency of sexual practice, the application of vaginal products and the history of sexually transmitted infections need to be considered to minimise risks and obtain a desirable VMB for transplantation [68]. For VMT to be a viable approach in the treatment of VVC, further randomised, placebo-controlled studies using large sample sizes should be carried out to confirm the effectiveness and durability of VMT, with standardised application routes, dosages and durations. Moreover, it is urgent for FDA to determine proper regulatory pathways regarding VMT and establish a framework to supervise the collection, transportation and screening of donated VMT material to prevent contamination and cross-contamination during manufacture [112]. In conclusion, VMT presents a promising approach to restore VMB balance. With improving diagnostic and detection ways to reduce potential pathogen transmission, VMT may act as an efficient alternative or complementary therapeutic strategy in the management of vaginal disorders, such as VVC, in the future.

### 4.5 Postbiotics may be a safer option in VVC treatment

Due to the potential risk of applying live microorganisms in neonates and immunosuppressed patients, there is an increasing interest in using postbiotics as novel therapeutic strategies for VVC [113]. Postbiotics refer to the formulation of inanimate microorganisms and/or soluble factors released from them, which directly or indirectly benefit host health [114]. Many available postbiotics contain inanimate strains belonging to the genera *Lactobacillus* or *Bifidobacterium,* with or without their metabolic compounds (e.g., enzymes, short-chain fatty acids, polysaccharides, biosurfactants, organic acids) secreted in CFS, which can be collected from their cell cultures [115]. Compared to probiotics, non-viable postbiotics are a safer option with a longer shelf life, easier storage, clearer chemical structures and more predictable mechanisms of action as they are unable to translocate from the intestinal cavity into the bloodstream [116]. Until now, postbiotics have been used not only in fermented functional food but also showed effects in preventing gastrointestinal disorders such as bloating and diarrhoea via maintaining intestinal microbiota homeostasis and reducing host inflammation as well as oxidative stress [117]. Due to anti-inflammatory, anti-oxidant and antimicrobial properties of postbiotics, multiple *in vitro* and *in vivo* studies have been conducted to evaluate the potential bioactivity of postbiotics to counteract fungal infections caused by *Candida* spp.

The CFS obtained from several *Lactobacillus* strains exerts inhibitory actions on *C. albicans* and NAC species. Sabbatini et al. (2021) indicated that *Lactobacillus-*derived CFS can suppress the growth and biofilm development of *C. albicans* isolated from women with VVC *in vitro* by interfering with the expression levels of adhesion (*ALS3*) and hyphae formation genes (*HWP1*; *CPH1*) [118]. In *in vivo* studies, prophylactic CFS injection restored the integrity of infected vaginal tissues in murine models infected with VVC [119]. Moreover, Parolin et al. (2021) designed a vaginal formulation containing hyaluronic acid (HA) and *L. crispatus* BC5 lyophilised CFS as a new therapeutic strategy for VVC, which is supported by the suppression of *C. albicans* growth after treatment [120]. The anti-*Candida* effects of CFS may mainly attribute to lactic acid and biosurfactants (BS). In particular, *Lactobacillus*-derived BS have attracted increased interest in the treatment of VVC due to their proven inhibition of adhesion, yeast-to-hyphae transition and biofilm formation of *Candida* species [[121], [122], [123]]. Other lactobacilli postbiotics, such as secreted proteins and SCFAs (acetate, butyrate and propionate), also demonstrated their role in improving vaginal epithelium integrity and restoring the balance between Th1 and Th2 lymphocytes during VVC, which may limit the tissue damage induced by *Candida* [124,125].

In this context, postbiotics with unique features may be safely used as supplements to live probiotics in the management of vaginal conditions such as VVC, without risks of acquiring antibiotic resistance genes and virulence factors [113]. However, additional placebo-controlled clinical trials should be designed and are necessary to determine the beneficial dosage, administration frequency and exact mechanisms of postbiotic interaction. Detailed regulation measures are also needed to ensure the biological characteristics and stability of postbiotics during the manufacturing process and delivery, with the purpose of eliminating the potential variability of postbiotics production.

### 4.6 Can prebiotics or synbiotics help relieve of VVC?

Prebiotics are substrates that selectively act on host microbes and have positive effects on host health, including non-digestible oligosaccharides (e.g., galactooligosaccharides [GOS] and fructooligosaccharides [FOS]), resistant starch, raffinose and inulin [126]. Prebiotics have showed their role in stimulating the metabolism of native *Bifidobacteria* and *Lactobacillus* spp. in the intestine, which reduced the risk of dysbiosis and related GI infections [126,127]. A major prebiotic, lactoferrin, exhibits antifungal activity against *Candida* species *in vitro* and in mice models with candidiasis [128]. The possible mechanisms involve the inhibition of morphological transition and biofilm formation of *Candida* [129,130]. Moreover, lactoferrin may also stimulate the antifungal activities of host cells by enhancing antimicrobial activities of macrophages and neutrophils, as well as the secretion of AMPs by epithelial cells [131,132]. Several studies also demonstrated a synergistic effect of lactoferrin combined with several antifungal agents (e.g., fluconazole) on *Candida* species by inhibiting hypha and biofilm formation in the early stage in a dose-dependent manner [133]. Other prebiotics (e.g., lactulose) also enhance vaginal *Lactobacillus* growth instead of *C. albicans*, providing evidence for the future application of prebiotics against VVC and the reduction of the need for antimicrobial agents in maintaining vaginal homeostasis [14].

Synbiotics were first proposed in 1995 and refer to a combination of prebiotics and probiotics that exert a synergistic effect [134]. Since properly selected prebiotics can improve the growth and metabolism of probiotic bacteria as well as their tolerance to environmental conditions, synbiotic products provide beneficial effects on host health through facilitating the survival and adhesion of beneficial microorganisms in the vagina. Moreover, synbiotics positively affect host health by leading to a higher concentration of advantageous metabolites such as SCFAs and ketones [135]. Synbiotic products containing a fusion of *Bifidobacteria* and/or *Lactobacillus* with FOS and/or inulin are most frequently used and have antibacterial, antiviral, antitumorigenic and immunomodulatory properties [136]. For example, García-Gamboa et al. (2022) proved that the synbiotic formulation of *L. paracasei* or *L. plantarum* in combination with inulin-type fructans resulted in a higher release of organic acid, which may be used in the prevention of VVC by significantly inhibiting virulent factors of *C. albicans* [137]. Similarly*,* numerous studies have evaluated the capability of a combination of bovine lactoferrin (BLF) and *Lactobacillus* to counteract VVC both *in vitro* and *in vivo*. Liao et al. (2019) demonstrated that counts of colony-forming units and the median colony size of *C. albicans* are decreased under co-cultivation with an *L. casei* strain that produced BLF [125]. In a murine VVC model, intravaginal application of *L. casei-*BLF resulted in a lower amount of vaginal discharge during VVC after a 5-day treatment and improved the host defense system through modulating cytokine (e.g., IL-17 and IL-23) secretion mediated by CD4^+^ T cells [125]. Moreover, the Respecta complex, containing two probiotic strains *L. acidophilus* and *L. rhamnosus* together with BLF, has been used to prevent bacterial vaginal infections through the normalisation of the Nugent score and a decrease in symptoms associated with vaginal dysbiosis, including irritation and discharge. Rosario et al. (2019) carried out a randomised clinical study involving 48 women (age 18–50 years) to investigate the effectiveness of the Respecta complex against VVC as a complementary treatment to topical clotrimazole. Compared with the placebo group, probiotic intervention alleviated VVC symptoms (58.4%) and lowered the recurrence rate (70.8%) at 3 and 6 months after treatment [138]. These results further support the potential application of synbiotics against VVC in the clinical practice.

Synbiotics have similar limitations as probiotics in terms of safety, especially when used in patients with immune deficiency. Moreover, the co-administration of variable probiotic species with a proper prebiotic and the lack of uniform subgroups regarding age, gender as well as lifestyle mode might contribute to controversial results. Since supplementation with prebiotics offers an effective, simple and convenient way to enhance probiotic metabolism, more large-scale RCTs should be carried out in humans to evaluate the cost-effectiveness, dose-response effects and optimal duration of administration [139].

## 5 Conclusions

The current mainstream therapy for VVC is still the use of antifungal medications, which remain critical challenges such as high recurrence rate and the appearance of drug-resistant *Candida* species. Therefore, it is urgent to explore alternative/complementary treatments in the clinical work. Probiotics are most widely studied and may play a more active role when used in combination with prebiotics or traditional antifungal drugs in the management of VVC, based on their strong immunomodulatory, antibiofilm and antifungal properties as well as abilities to restore normal flora communities in the vagina. Nevertheless, the safety of probiotics and health status of VVC patients should be strictly considered before administering viable probiotics due to strain-specific effects and the possibility of rare bacteraemia in women with immune deficiency. Postbiotics are considered to counteract VVC due to their anti-inflammatory, anti-oxidant and antimicrobial properties. The advantage of postbiotics is that they are more safe without the risk of applying live microorganisms. VMT also presents great potential in the treatment of VVC via constructing a new vaginal micro-ecological balance, but research in this area is still in its infancy and lacks sufficient *in vivo* and clinical data to support the feasibility of VMT. In conclusion, although various experimental studies support the administration of probiotics or synbiotics, postbiotics and VMT may exert a protective effect against VVC, we need more high-quality, large-scale randomised controlled trials to further explore the underlying mechanisms and confirm the safety and effectiveness of these new interventions.

## Funding

This work was supported by grants from the 10.13039/501100001809National Natural Science Foundation of China (no. 82060638) and ‘Double 10 −Thousand Plan’ of Jiangxi Province (Innovation and Technology Professionals as the High−End Talent), The National Natural Science Foundation of China (grant no.82260298).

## Data availability

No data was used for the research described in the article.

## Ethical approval

Ethical approval and consent of patients to participate is not applicable to our study.

## CRediT authorship contribution statement

**Yufei Wang:** Writing – original draft. **Zhaoxia Liu:** Writing – review & editing. **Tingtao Chen:** Writing – review & editing.

## Declaration of competing interest

The authors declare that they have no known competing financial interests or personal relationships that could have appeared to influence the work reported in this paper.
