# Supplementary material for: Vaginal microbiota: Potential targets for vulvovaginal candidiasis infection
Source: Heliyon. 2024 Mar 2;10(5):e27239. doi: 10.1016/j.heliyon.2024.e27239 (PMC10923723; doi:10.1016/j.heliyon.2024.e27239)
Supplement: Multimedia component 1 [file mmc1.pdf]

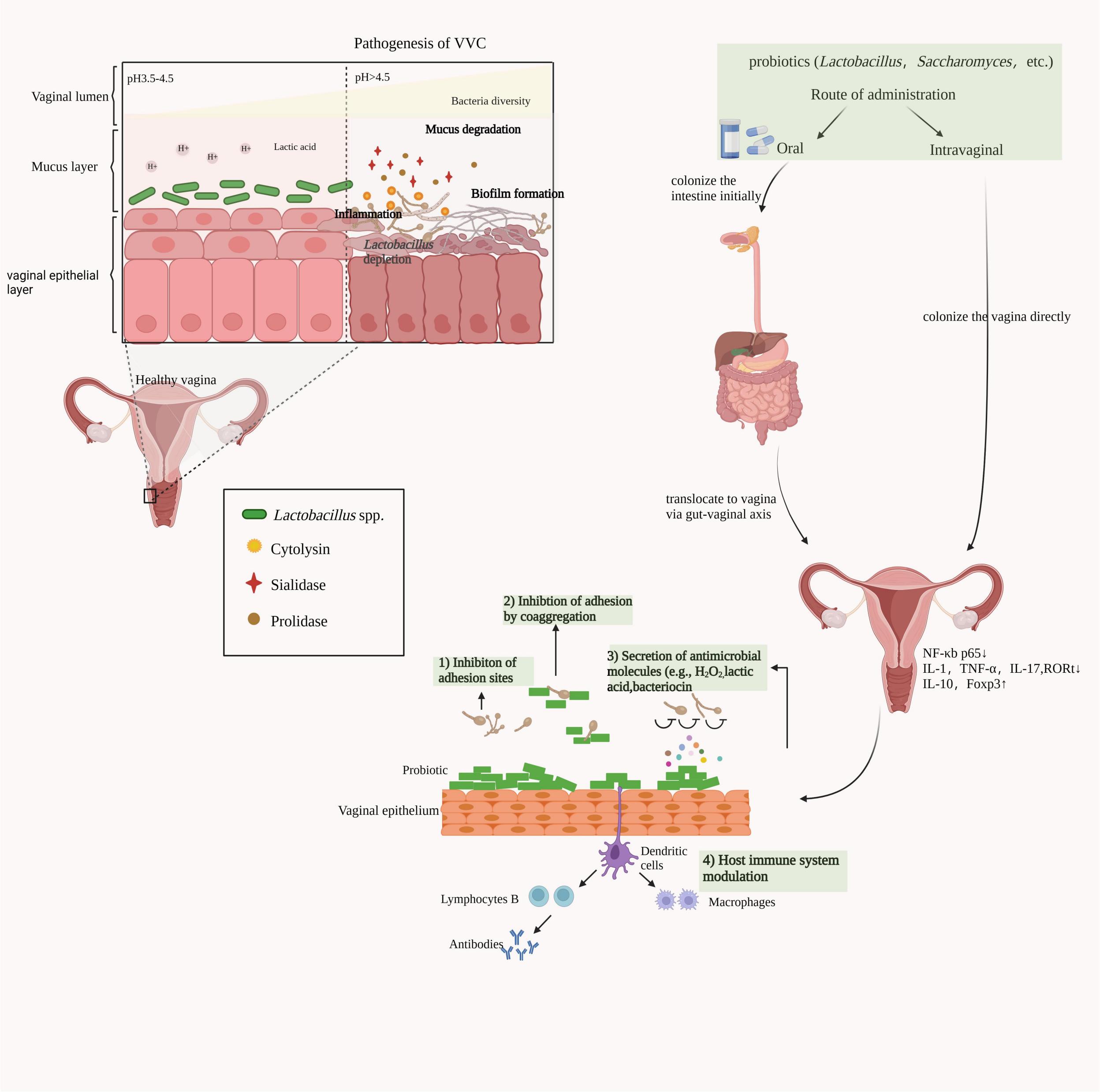


Fig.1 Pathogenesis of VVC and mechanisms of probiotic. The *Lactobacillus* depletion and the overgrowth of *Candida* species contribute to pathogenesis of VVC. Probiotics can be administered orally or intravaginally. The postulated mechanisms of probiotic include (1) reduction of the initial adhesion and colonization of *Candida* species; (2) competition of adhesion sites and nutrients on mucosal surface; (3) production of secondary metabolites with antimicrobial activity and (4) modulation of the immune response.
