# Supplementary material for: Vaginal microbiota: Potential targets for vulvovaginal candidiasis infection
Source: Heliyon. 2024 Mar 2;10(5):e27239. doi: 10.1016/j.heliyon.2024.e27239 (PMC10923723; doi:10.1016/j.heliyon.2024.e27239)
Supplement: Multimedia component 2 [file mmc2.pdf]

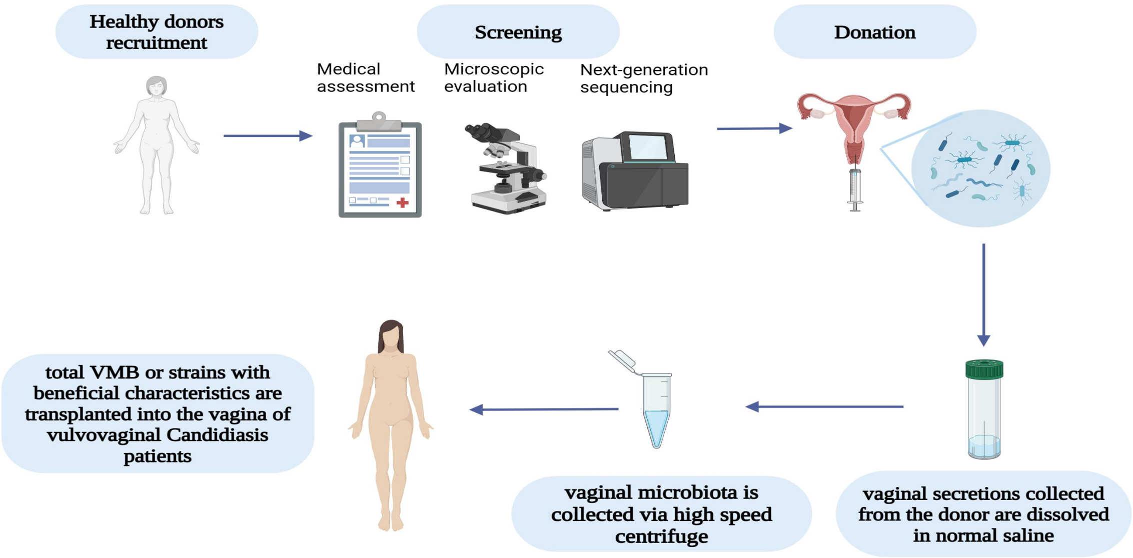

Fig.2 Technological procedure of VMT. First, choose the healthy donor and collect her vaginal secretions after careful screening. Separate the total VMB from the vaginal secretion through the centrifugation. Then vaginally inoculated VMB suspension into patients with vaginal dysbiosis. The probiotics from vaginal secretions of donors can also be screened, and those strains with beneficial characteristics are mixed together and transplanted into the vagina of patients.
