# Supplementary material for: Vaginal microbiota: Potential targets for vulvovaginal candidiasis infection
Source: Heliyon. 2024 Mar 2;10(5):e27239. doi: 10.1016/j.heliyon.2024.e27239 (PMC10923723; doi:10.1016/j.heliyon.2024.e27239)
Supplement: Multimedia component 3 [file mmc3.docx]

| Brief summary of clinical studies using *Lactobacillus*-based probiotics | | | |
| --- | --- | --- | --- |
| Included study | Probiotic strains tested | Intervention doses and duration | Results |
| The role of single strain in the prevent and treatment of VVC | | | |
| Hilton et al.^63^ | *L.* *acidophilus* | Intake of 8 ounces/day yogurt  containing *L.* *acidophilus* for 6 months then no yogurt for the next 6 months | Women in yogurt  group showed a  decrease in both  candidal colonization  (0.84 +/- 0.90 vs 3.23  +/- 2. 17) and infection  (0.38 +/- 0.51 vs 2.54  +/- 1.66) compared  to those in control  group. |
| Williams et al.^64^ | *L.* *acidophilus* | weekly intravaginal application of *L. acidophilus* or weekly intravaginal application of clotrimazole tablets (100mg) | The estimated median time to first episode VVC was longer for clotrimazole (p = .03, log rank test) and *L.acidophilus* (p = .09, log rank test) compared with placebo. |
| The role of probiotic combinations in the prevent and treatment of VVC | | | |
| Reid et al.^70^ | *L.* *rhamnosus* GR-1 and  *L.reuteri* RC-14 | Ingestion of a freeze-dried probiotic capsule (>10^9^ per strain), or calcium carbonate placebo daily for 60 days. | Women in probiotic  group (37%) showed  a higher colonization  of vaginal  lactobacilli at day 28  (P=0.08) and day 60  (P=0.05), and a  higher depletion in  yeast at day 28  compared those in the  control group (13%) |
| Martinez et al.^71^ | *L.* *rhamnosus* GR-1 and  *L.reuteri* RC-14 | Ingestion of a single dose of  fluconazole (150mg) plus  either two oral probiotic capsules or placebo once  daily (every morning) for 4  weeks | At 4 weeks, women  treated by probiotic  showed remarkably  less VVC symptoms  such as vaginal  discharge ( 10.3% vs  34.6%; P=0.03) and  lower number of  cultured yeasts  ( 10.3% vs 38.5%;  P=0.014). |
| Ehrstrom, S., et al.^78^ | probiotic group (*L* *gasseri* LN40, *L.* *fermentum* LN99, *L.* *casei* subsp. *rhamnosus* LN113 and *P.* *acidilactici* LN23) | clotrimazol 200 mg vaginal tablets for 3 consecutive nights, followed by vaginal administration of either capsules containing probiotic bacteria strains or placebo capsules twice daily, for five consecutive days. | 93% of the women receiving probiotics were cured 2–3 days after administration (placebo: 83%), and 78% after one menstruation (placebo: 71%) (ns). The intervention group experienced less malodorous discharge 2–3 days after administration (p = 0.03) and after the second menstruation (p = 0.04), compared with placebo. |

|  |  |  | vs 0%), higher cure  rate (93% vs 83%)  and less malodorous  discharge (p=0.03). |
| --- | --- | --- | --- |
| Kovachev et al.^79^ | probiotic group (*L.*  *acidophilus*, *L.* *rhamnosus*, *S.* *thermophilus*, and *L.* *delbrueckii* subsp. *bulgaricus)* | The first group was  administered 150 mg  fluconazole and a single 600  mg fenticonazole once. The  second group received the  same conventional thrapy,  followed by using vaginal  probiotics on the fifth day after azole treatment. | Women in the second  group reported less  clinical complaints  (79.7 % vs 31. 1 % )  and improved  colonization of  vaginal lactobacilli  (95.2 % vs 93.7 %)  compared to those in  the first group. |
| Davar et al.^80^ | probiotic group (*L. acidophilus, B. bifidum, B. longum*) | single dose of 150 mg of fluconazole tablet, plus oral probiotic tablet or placebo twice a day for a 10-day period | the 6-month recurrence in the control group was eleven (35.5%) and in the research group was two (7.2%). The results from Fisher’s exact test for the value *p* = 0.01 and OR 0.14 95 % CI (0.028–0.7) showed significant recurrence in the placebo group. |
| Vahedpoor et al.^81^ | vaginal probiotic (*L.* *acidophilus*, *L.*  *plantarum*, *L.* *rhamnosus*, *L.*  *Gasseri*) and oral  probiotic (  containing *L.* *acidophilus*, *L.*  *plantarum*, *L.fermentum*, *L.*  *gasseri*) | The first group was received  an oral 150mg fluconazole and vaginal probiotic 1×109 CFU/day for  each for 14 nights and oral  probiotic for 30 days . The  second group was received an  oral 150mg fluconazole and  vaginal and oral placebo for  14 nights and 30 days  respectively. | Women in probiotics  supplementation  group showed a  significant reduction  in VVC symptoms  (46.9% vs. 68.4%,  P=0. 184) and fewer  presence of positive  culture of yeast. (44%  vs. 56%, P=0.291) |

Table1 Brief summary of interventions in included clinical studies using *Lactobacillus*-based probiotics
